# Effect of Er:YAG laser enamel conditioning and moisture on the microleakage of a hydrophilic sealant

**DOI:** 10.1007/s10266-017-0323-4

**Published:** 2017-12-13

**Authors:** Zeynep Aslı Güçlü, Andrew Paul Hurt, Nazmiye Dönmez, Nichola Jayne Coleman

**Affiliations:** 1 Department of Paediatric Dentistry, Faculty of Dentistry, Erciyes University, Kayseri, Turkey; 2 Department of Pharmaceutical, Chemical and Environmental Sciences, Faculty of Engineering and Science, University of Greenwich, Chatham Maritime, Kent, UK; 3 Department of Dental Diseases and Treatment, Bezmiâlem Vakif University, Istanbul, Turkey

**Keywords:** Hydrophilic sealant, Laser, Microleakage, Moisture, Saliva

## Abstract

For a given sealant, successful pit and fissure sealing is principally governed by the enamel conditioning technique and the presence of moisture contamination. A new generation of hydrophilic resin sealants is reported to tolerate moisture. This study investigates the impact of Er:YAG laser pre-conditioning and moisture contamination on the microleakage of a recent hydrophilic sealant. Occlusal surfaces of extracted human molars were either acid etched (*n* = 30), or successively lased and acid etched (*n* = 30). Ten teeth from each group were either air-dried, water-contaminated, or saliva-contaminated prior to sealing with UltraSeal XT^®^ hydro™. Samples were inspected for penetration of fuchsin dye following 3000 thermocycles between 5 and 50 °C, and the enamel–sealant interfaces were observed by scanning electron microscopy (SEM). Significant differences in microleakage were evaluated using the Mann–Whitney *U* test with Bonferroni adjustment (*p* = 0.05). Laser pre-conditioning significantly reduced dye penetration irrespective of whether the enamel surface was moist or dry. Microleakage of water-contaminated acid etched teeth was significantly greater than that of their air-dried or saliva-contaminated counterparts. SEM analysis demonstrated good adaptation in all groups with the exception of water-contaminated acid etched teeth which exhibited relatively wide gaps. In conclusion, this hydrophilic sealant tolerates the presence of saliva, although water was found to impair its sealing ability. Laser pre-conditioning significantly decreases microleakage in all cases.

## Introduction

A wide range of light-cured resin-based sealants is commercially available for the isolation and defence of occlusal surfaces of molars and premolars [1, 2]. The majority of these pit and fissure sealants are hydrophobic materials that bond to etched air-dried enamel via micromechanically interlocking tags. Moisture contamination compromises the quality of adhesion at the sealant–enamel interface, and is a common challenge encountered in paediatric dentistry where patient compliance is low [3]. To address this problem, hydrophilic sealants have appeared on the market during the past decade which are specifically designed to be placed on moist enamel [3, 4].

Current research has indicated that the application of laser ablation as an adjunct to traditional phosphoric acid etching may improve the adhesion, adaptation, retention, and resistance to microleakage of resin-based sealants [3, 5]. Recent clinical and in vitro studies support the use of laser ablation prior to acid etching [3–6], although these findings are not unanimously confirmed [7–9] In practice, the effectiveness of laser pre-conditioning appears to depend on many factors relating to the rheological and physicochemical properties of the particular sealant, lasing parameters and operator technique.

The principal objectives of this study were to investigate the impact of laser pre-conditioning and moisture contamination on the resistance to microleakage of the new hydrophilic self-adhesive sealant, UltraSeal XT^®^ hydro™ (Ultradent Products, USA) [10], in vitro. The occlusal surfaces of extracted human molars were either acid etched (*n* = 30), or successively lased and acid etched (*n* = 30). Ten teeth from each group were then either air-dried, water-contaminated, or saliva-contaminated prior to sealing with UltraSeal XT^®^ hydro™. The samples were inspected for microleakage using fuchsin dye penetration following 3000 thermocycles between 5 and 50 °C. Microleakage scores were analysed using the Kruskal–Wallis test and Mann–Whitney *U* test with Bonferroni adjustment. The following null hypotheses were tested (*p* = 0.05): (1) there is no difference in microleakage among traditionally acid etched teeth that are either air-dried, water-contaminated or saliva-contaminated; (2) enamel pre-conditioning by laser ablation prior to acid etching has no impact on resistance to microleakage; and (3) the presence of moisture does not influence microleakage of teeth that have been successively lased and acid etched.

During this study, the nature of the enamel–sealant interface was also observed by scanning electron microscopy (SEM) with energy-dispersive X-ray (EDX) analysis.

## Materials and methods

Ethical approval for this project was obtained on the 1st of October 2014 by the Ethical Committee of Bezmiâlem Vakif University (reference number 71306642/050-01-04/282). The study was performed in accordance with the ethical standards laid down in the 1964 Declaration of Helsinki and its later amendments.

### Sample preparation

Sixty sound extracted human molar teeth were obtained from patients with orthodontic or periodontal problems who had provided their written informed consent. The teeth were debrided with manual scaling instruments, cleaned with bristle brush and pumice paste and stored in distilled water for up to 5 days. The teeth were then randomly divided into 6 groups, as indicated in Table 1. All samples were prepared by the same operator.

**Table 1:** Experimental groups

Group	*n*	Enamel pre-treatment	Surface condition
I	10	Acid etch	Dry
II	10	Acid etch	Moist with water
III	10	Acid etch	Moist with saliva
IV	10	Lase and acid etch	Dry
V	10	Lase and acid etch	Moist with water
VI	10	Lase and acid etch	Moist with saliva

The occlusal surfaces of Group I teeth were acid etched for 20 s with 35% phosphoric acid gel (UltraSeal XT^®^ hydro™, Ultradent Products, USA), rinsed and air-dried. The UltraSeal XT^®^ hydro™ sealant (Ultradent Products, USA) was then applied, according to the manufacturer’s instructions and light cured for 20 s with a BA Optima 10 curing light (BA International Ltd., Northampton, Northamptonshire, UK).

Group II and III teeth were acid etched, rinsed and air-dried (as outlined above). The occlusal surfaces were then contaminated with 0.1 cm^3^ of either distilled water (Group II) or fresh saliva (Group (III) for 20 s using a dropping pipette and then lightly dried using a cotton pellet which was applied for 5 s, prior to the application of the sealant [3].

Laser conditioning of the occlusal surfaces of Group IV teeth was carried out using an Er:YAG laser system (LightWalker^®^, Fotona, Slovenia) operating at a wavelength of 2940 nm, a power output of 1.2 W, pulse energy of 120 mJ and a frequency of 10 Hz. Laser ablation was carried out using a 600 μm diameter sapphire tip with a beam spot size of 0.63 mm^2^, energy density of 19 mJ cm^2^ at a working distance of 8 mm at an angle of 90° under water cooling at 50 cm^3^ min^−1^. The teeth were then rinsed with water, air-dried and acid etched, as described above, prior to the application and curing of the UltraSeal XT^®^ hydro™ sealant. Groups V and VI teeth were sequentially lased and acid etched (as outlined above). The occlusal surfaces of Group V were contaminated with distilled water and Group VI teeth were contaminated with fresh saliva prior to the application of the sealant.

### Microleakage analysis

Immediately after sealing, the teeth were placed in distilled water at 37 °C for 24 h and then thermocycled 3000 times between 5 and 55 °C with a transfer time of 10 s and a dwell time of 30 s [4, 5]. Subsequent microleakage was assessed via dye penetration. The teeth were coated with nail varnish, leaving a 2 mm window around the sealant, and the roots were embedded in an acrylic resin cylinder (Meliodent, Bayer Dental, UK). The teeth were then placed in 0.5% basic fuchsin dye solution for 24 h. Following immersion, the teeth were rinsed under running tap water for 5 min to remove excess dye and sectioned in the buccolingual direction using a water-cooled diamond saw to obtain three slices. Each of the tooth sample slices was then examined by two blind investigators under a stereomicroscope (SMZ 800, Nikon, Japan) at 20× magnification. Microleakage scoring criteria are listed in Table 2 [11].

**Table 2:** Microleakage scoring criteria

Score	Definition
0	No dye penetration
1	Dye penetration up to half of the fissure
2	Dye penetration beyond half of the fissure without total involvement
3	Dye penetration to the sealant base

Inter-examiner reproducibility was analysed with the kappa statistic. Median differences among the microleakage data for each of the groups were compared using the Kruskal–Wallis test (*p* = 0.05). Significant differences were evaluated using the Mann–Whitney *U* test with Bonferroni adjustment (*p* = 0.05).

### Scanning electron microscopy

Scanning electron microscopy was carried out on the central slices of the sectioned teeth using uncoated samples attached to carbon tabs on a JEOL JSM-5410 LV electron microscope with an Oxford Instruments X-MaxN EDX detector in low vacuum mode. All back-scattered electron images and EDX maps were obtained with an accelerating voltage of 20 kV at a working distance of 20 mm.

## Results

### Microleakage analysis

The distributions of microleakage scores for each experimental group are listed in Table 3. An inter-examiner kappa statistic of 0.90 was obtained for the microleakage evaluation which indicates high reproducibility.

**Table 3:** Microleakage scores as functions of moisture and enamel conditioning

Score	0	1	2	3	Debonded	Median	Significance*
Group I	10	12	4	4	0	1	a
Group II	0	6	6	17	1	3	b
Group III	2	16	8	4	0	1	a
Group IV	20	7	0	2	0	0	c
Group V	17	9	1	3	0	0	c
Group VI	16	6	6	2	0	0	c

* Different letters indicate significant differences among the groups (*p* < 0.05), whereas the same letters indicate no significant differences (*p* > 0.05)

No significant difference in marginal leakage was found between the separately acid etched groups that were either air-dried (Group I) or saliva-contaminated (Group III) (*p* = 0.09), although the number of samples that exhibited no visible microleakage was greater in the former case. Conversely, Group II teeth that were acid etched and water-contaminated exhibited significantly higher microleakage than those of both Group I (*p* < 0.001) and Group III (*p* < 0.001). Hence, the null hypothesis that there is no difference in microleakage between acid etched samples that were air-dried or moist is rejected in the case of water contamination, but retained in the case of saliva contamination. None of the samples in Group II was entirely resistant to dye penetration, with over half of the specimens in this group exhibiting maximum penetration to the sealant base. Furthermore, the sealant partially detached (i.e. ‘debonded’) from the central section of one of the samples in the water-contaminated group prior to inspection for microleakage.

In all cases, irrespective of whether the teeth were air-dried or contaminated with either water or saliva, laser ablation as an adjunct to acid etching was found to significantly improve resistance to microleakage (*p* < 0.05). The median and modal microleakage scores for Groups IV, V, and VI were zero, indicating that the majority of the lased samples completely resisted dye penetration. Among these groups, there were no significant differences in microleakage irrespective of the presence or absence of either water or saliva. Therefore, the null hypothesis that enamel pre-conditioning by laser ablation prior to acid etching has no impact on microleakage is rejected across all groups. Conversely, the null hypothesis that water or saliva contamination does not influence microleakage of sealed teeth that have been consecutively lased and acid etched is retained.

### Scanning electron microscopy

**Fig. 1:**
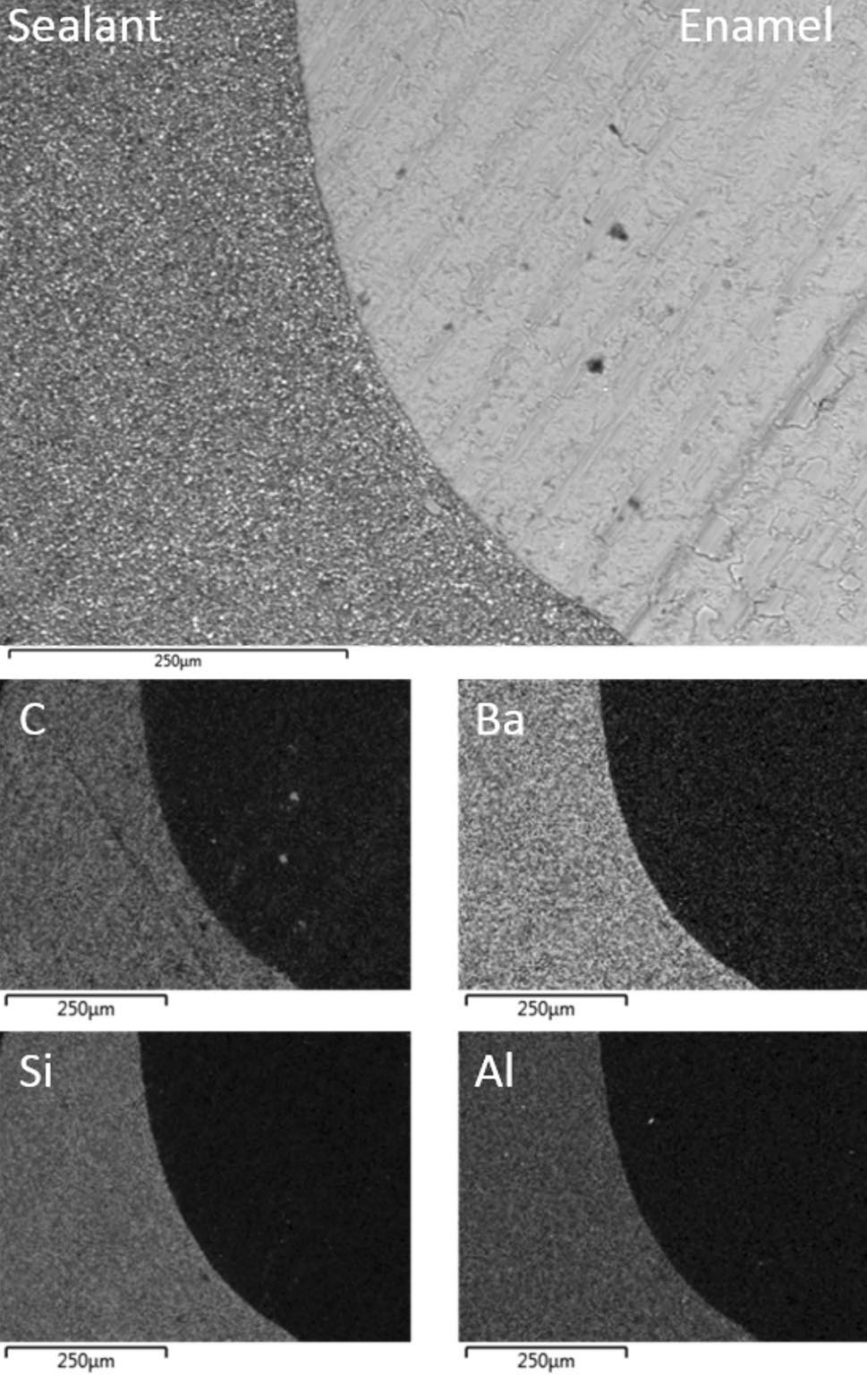
Back-scattered SEM image of UltraSeal XT^®^ hydro™ in contact with Group I air-dried acid etched enamel; and corresponding EDX maps of carbon, barium, silicon and aluminium

The back-scattered electron image of the enamel–sealant interface of a random Group I acid etched air-dried tooth is shown in Fig. 1 along with the corresponding EDX elemental maps of carbon, barium, silicon and aluminium. In this group, the sealant was well adapted to the acid etched air-dried enamel and the barium-, aluminium- and silicon-bearing inorganic filler phases remained homogeneously distributed throughout the organic resin matrix.

**Fig. 2:**
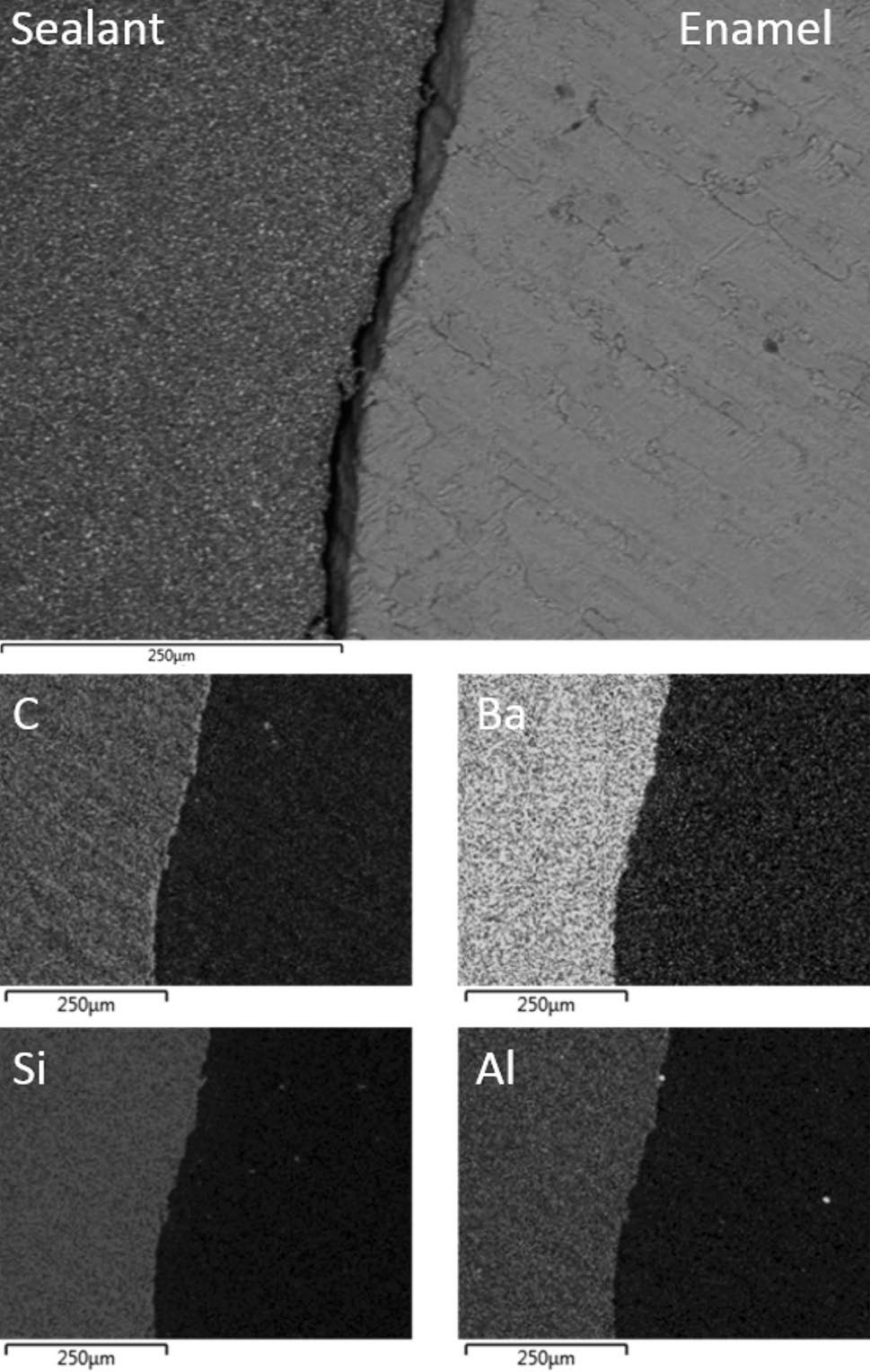
Back-scattered SEM image of UltraSeal XT^®^ hydro™ in contact with Group II water-contaminated acid etched enamel; and corresponding EDX maps of carbon, barium, silicon and aluminium

**Fig. 3:**
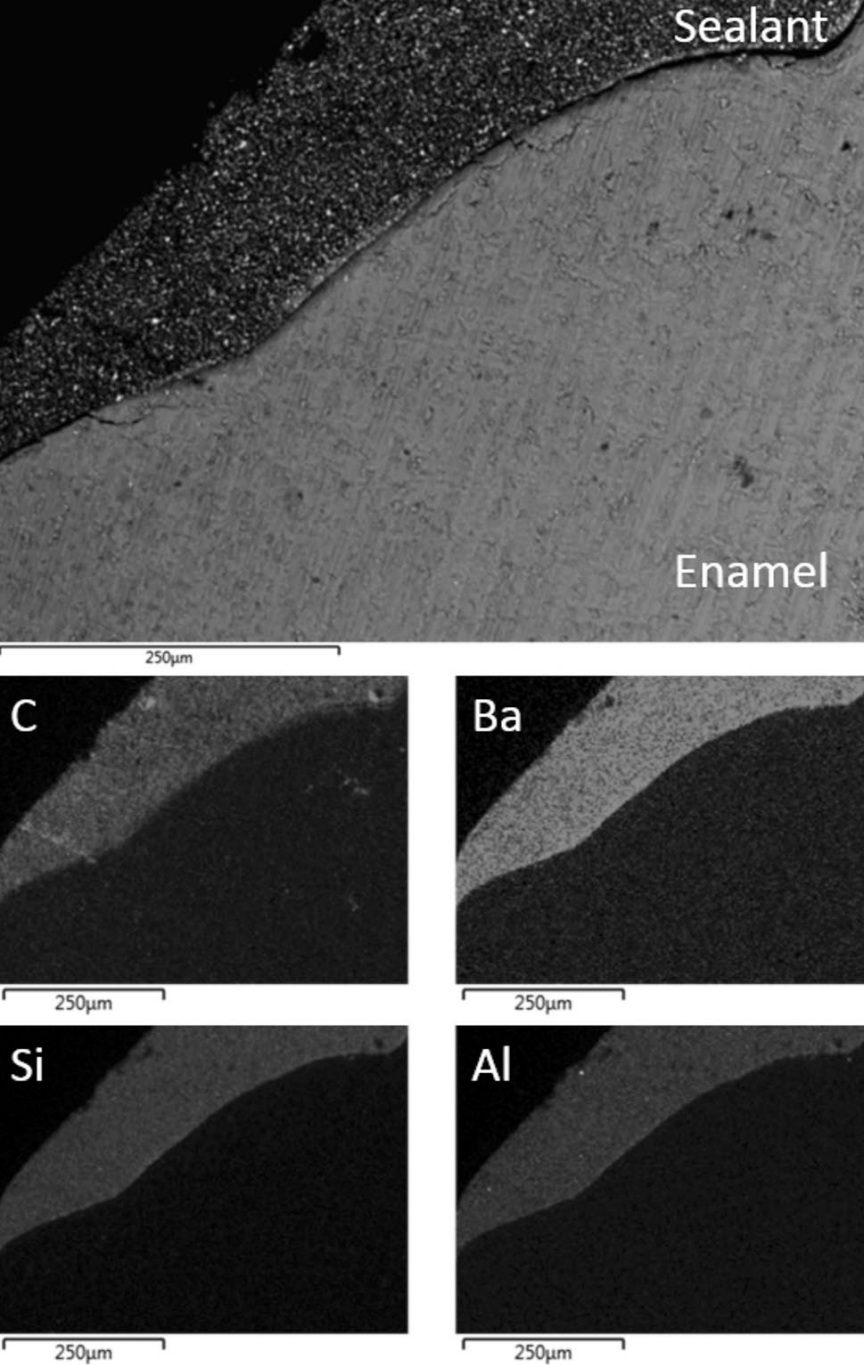
Back-scattered SEM image of UltraSeal XT^®^ hydro™ in contact with Group III saliva-contaminated acid etched enamel; and corresponding EDX maps of carbon, barium, silicon and aluminium

SEM analysis indicated that Group II acid etched water-contaminated samples were characterised by relatively wide gaps of several tens of microns between the sealant and the enamel (Fig. 2). It is likely that the low vacuum pressure of approximately 15 Pa encountered in the electron microscope caused the poorly adapted sealant of the water-contaminated samples to detach from the enamel. On the contrary, the sealant was generally observed to be in intimate contact with the saliva-contaminated enamel of the Group III teeth, with occasional margins of a few microns (Fig. 3). The inorganic filler phases were seen to be uniformly distributed throughout the organic resin matrix of the sealant in Groups II and III.

**Fig. 4:**
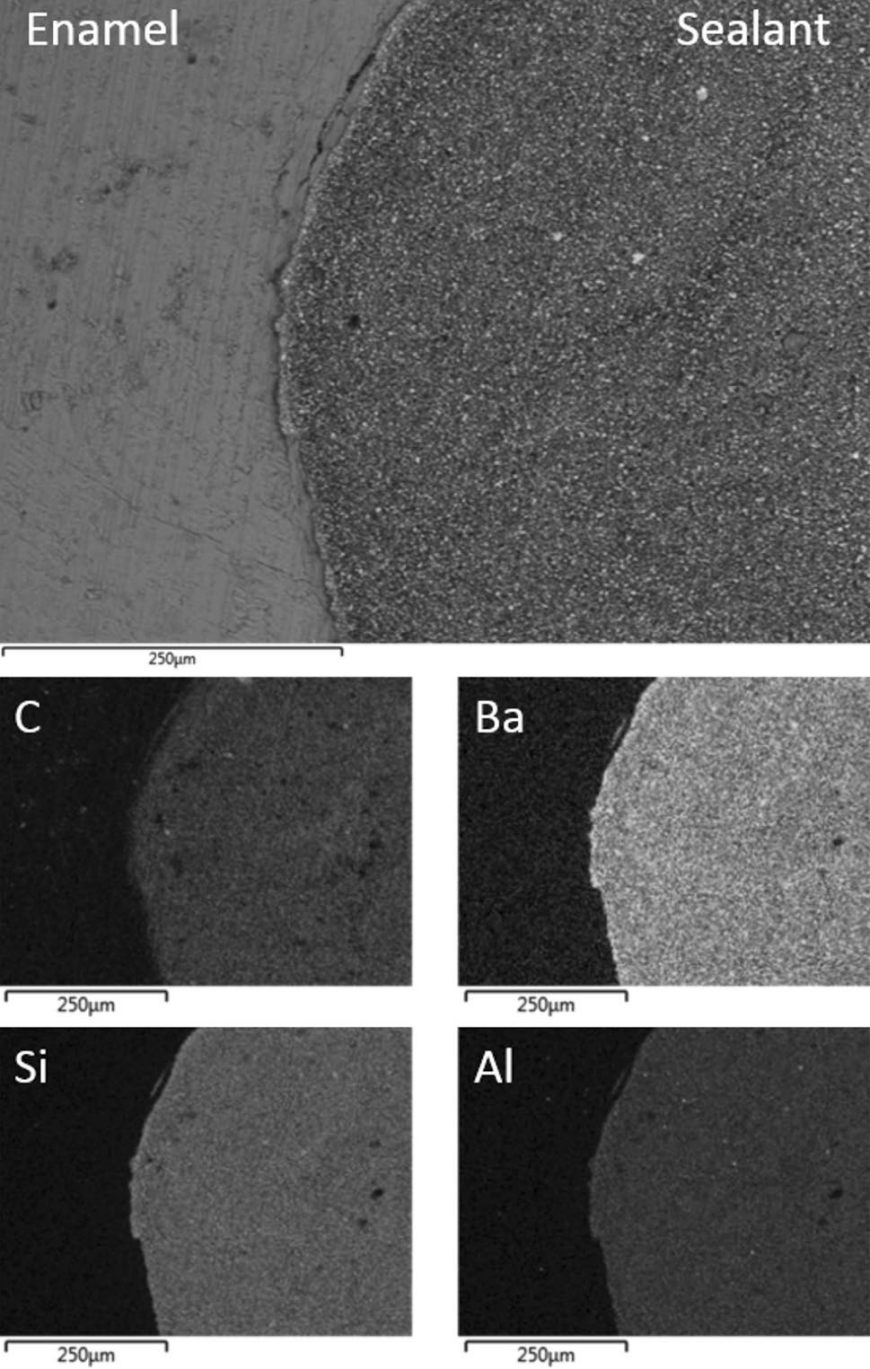
Back-scattered SEM image of UltraSeal XT^®^ hydro™ in contact with Group IV air-dried etched and lased enamel; and corresponding EDX maps of carbon, barium, silicon and aluminium

**Fig. 5:**
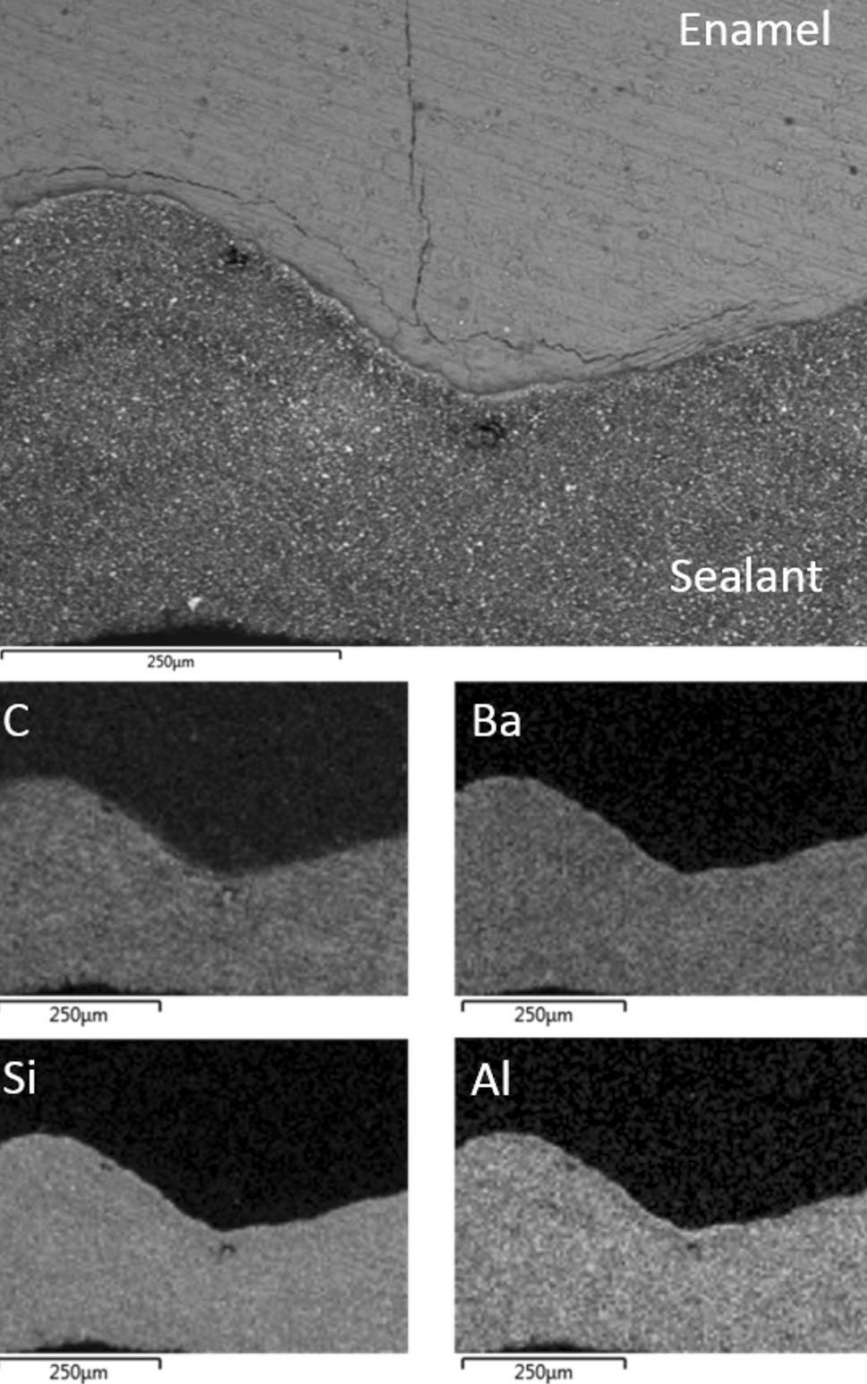
Back-scattered SEM image of UltraSeal XT^®^ hydro™ in contact with Group V water-contaminated etched and lased enamel; and corresponding EDX maps of carbon, barium, silicon and aluminium

**Fig. 6:**
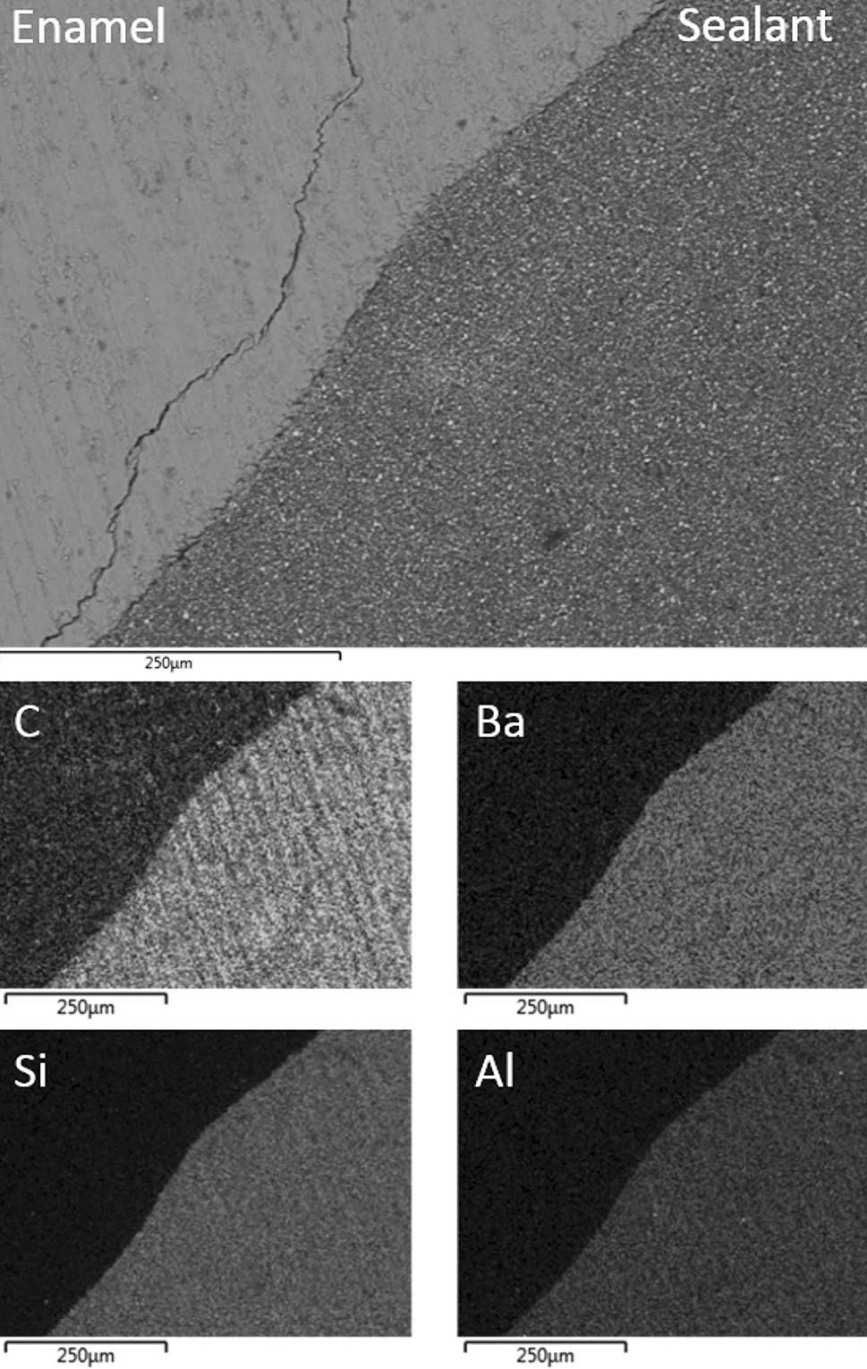
Back-scattered SEM image of UltraSeal XT^®^ hydro™ in contact with Group VI saliva-contaminated etched and lased enamel; and corresponding EDX maps of carbon, barium, silicon and aluminium

Representative back-scattered electron micrographs of the interfaces of the sealant and the laser pre-conditioned enamel of Groups IV, V, and VI are shown in Figs. 4, 5, and 6, respectively. In all cases, the sealant was observed to be in direct contact with the enamel. These samples also exhibited regions of sub-surface enamel cracking at depths of up to 150 μm from the interface. Some zoning of the inorganic filler particles at the enamel–sealant interface was noted for samples in Groups IV and V (Figs. 4, 5, respectively), although the filler remained homogeneously distributed in the Group VI specimens. The increased concentration of the filler at the enamel surface is attributed to the enhanced surface roughness of the lased enamel [4, 5], which appears to be mitigated by the lubricating effect of saliva.

## Discussion

UltraSeal XT^®^ hydro™ is a recently marketed moisture-tolerant, self-adhesive, acrylate-based pit and fissure sealant which has been developed by Ultradent Products, USA [4]. It is highly filled with 53% inorganic phases which are incorporated to confer radiopacity and to improve wear-resistance. UltraSeal XT^®^ hydro™ is reported to ‘chase’ moisture into the pits and fissures, thus eliminating moisture-related failures in adaptation and retention which are common to hydrophobic sealant materials [10].

Since this is a new material, at the present time, only one report could be located in the scientific literature concerning the relative sealing performance of UltraSeal XT^®^ hydro™ on air-dried or saliva-contaminated primary second molars [12]. This research confirms the manufacturer’s claim that surface contamination by saliva does not affect the sealing ability of this material; although, the impact of water contamination is not addressed in the study. Our present results also confirm that, after 3000 thermocycles between 5 and 55 °C, there is no difference in the extent of dye penetration when UltraSeal XT^®^ hydro™ is placed on conventionally etched enamel that is either air-dried or saliva-contaminated. To best identify any differences in sealing ability among the various experimental groups, our microleakage study employed relatively aggressive thermocycling parameters and 0.5% basic fuchsin solution which is considered the most effective dye for revealing microleakage [5].

Conversely, our results indicate that UltraSeal XT^®^ hydro™ does not effectively tolerate contamination by water to the same extent that it tolerates saliva. In the case of water contamination, wide gaps between the sealant and enamel were noted in all corresponding SEM images, and the relative resistance to dye penetration was diminished.

The direct clinical relevance of in vitro microleakage tests is questionable, although it is generally agreed that they can provide useful information on a dental sealant’s capacity to maintain good marginal adaptation to prevent bacterial ingress. The potential clinical significance of this study is that caution should be applied when considering the nature of the moisture contamination present at the time of placement and sealing with UltraSeal XT^®^ hydro™, as, in this respect, a discrepancy in performance has been identified between water and saliva.

Recent reports by the authors [4, 5] have demonstrated that teeth treated with a successive combination of Er:YAG laser irradiation and acid etching prior to sealing with UltraSeal XT^®^ hydro™ showed significantly lower microleakage scores than those that were exclusively acid etched. These studies included only air-dried enamel and did not consider the impact of moisture contamination on sealing ability. From the results obtained in our present study, it appears that laser pre-conditioning prior to acid etching improves resistance to microleakage irrespective of whether the enamel is air-dried or contaminated with either water or saliva. These findings concur with those of a similar study on another commercial hydrophilic sealant (Embrace WetBond™, Pulpdent/Gaba, USA) which demonstrated significant improvements in resistance to microleakage for laser pre-conditioned enamel which was contaminated with either water or saliva [3].

There is, currently, no universal agreement regarding the potential benefits of laser pre-conditioning on the retention and microleakage of self-adhesive resin-based fissure sealants. For example, studies by Ciucchi et al. [7], Borsatto et al. [8] and Youssef et al. [13] report no significant differences in hydrophobic sealant microleakage between teeth that were exclusively acid etched and those that were consecutively Er:YAG laser ablated and acid etched. It is speculated that highly viscous sealants adapt poorly to the enhanced roughness of lased enamel, which may account for the observed discrepancies in the literature.

A further consideration associated with the application of laser-pre-conditioning is the occurrence of sub-surface enamel cracking that has been noted in this and previous studies [4, 5, 14]. The presence of sub-surface microcracking is not visible to the clinician, and is also not readily observed in vitro using light microscopy, so the reported incidences are limited to the few studies in which scanning electron microscopy has been employed. At this point in time, the significance of laser-induced microcracking with respect to the longevity of the sealant and the ongoing welfare of the tooth has not yet been determined.

## Conclusions

This study concerns the impact of Er:YAG laser pre-conditioning and moisture contamination on the microleakage of a recent hydrophilic sealant (UltraSeal XT^®^ hydro™). No significant difference was observed in microleakage of acid etched teeth that were either air-dried or saliva-contaminated; unlike their water-contaminated counterpart which exhibited more extensive dye penetration. Laser pre-conditioning prior to conventional acid etching significantly increased resistance to microleakage irrespective of whether the enamel surface was moist or dry. Scanning electron microscopy (SEM) demonstrated good adaptation in all cases with the exception of water-contaminated acid etched teeth which exhibited relatively wide gaps between the sealant and enamel. SEM analysis also revealed sub-surface cracking of the enamel of teeth subjected to laser ablation.
